# Sexual Double Standards: Contributions of Sexual Socialization by Parents, Peers, and the Media

**DOI:** 10.1007/s10508-021-02088-4

**Published:** 2021-11-09

**Authors:** Joyce J. Endendijk, Maja Deković, Helen Vossen, Anneloes L. van Baar, Ellen Reitz

**Affiliations:** Child and Adolescent Studies, Utrecht University, Heidelberglaan1, P.O. Box 80140, 3508 TC Utrecht, The Netherlands

**Keywords:** Sexual double standards, Social norms, Adolescence, Socialization

## Abstract

(Hetero)sexual double standards (SDS) entail that different sexual behaviors are appropriate for men and women. There is large variation in whether people endorse SDS in their expectations about the sexual behavior of women and men (i.e., SDS-norms). To explain these individual differences, we examined associations between SDS-norms of Dutch adolescents (aged 16–20 years, *N* = 566) and what parents, peers, and the media teach adolescents about appropriate sexual behavior of boys and girls (i.e., SDS-socialization). Adolescents completed an online survey at school. Regarding SDS-socialization, more traditional SDS-norms conveyed by the media and peers, but not of parents, and less perceived sexual activity of female peers, were associated with more traditional SDS-norms. Only for boys, exposure to sexy girls/women on social media and sexual music videos of female artists were associated with more traditional SDS-norms. Thus, SDS-socialization by peers and the media and opposite gender models (for boys) are important in light of adolescents’ SDS-norms.

## Introduction

Women and men are often held to different standards of appropriate behavior (Foschi, 2000; Prentice & Carranza, 2002). A well-known example is the (hetero) sexual double standard (SDS), in which different sexual behaviors are expected of, and valued for, men and women (Emmerink et al., 2016; Zaikman & Marks, 2017). Traditionally, men/boys are expected to be sexually active, dominant, and the initiator of (hetero) sexual activity, whereas women/girls are expected to be sexually reactive, submissive, and passive. Moreover, traditionally men are granted more sexual freedom than women. As a consequence, women and men are treated differently when they show the same sexual behaviors. For example, slut-shaming is experienced by 50% of girls, compared to 20% of boys (Hill & Kearl, 2011). Traditional expectations about the sexual behavior of men and women (i.e., SDS-norms) are associated with gender differences in sexual risk behavior, specifically, with more sexual partners for men, and more reluctance to request or insist on condom use for women (Lefkowitz et al., 2014). SDS-norms have also been linked to the fact that most perpetrators of sexual coercion and violence are men, whereas most of their victims are women (Shen et al., 2012). Furthermore, concepts related to SDS-norms, such as gender role adherence in intimate relations (Sanchez et al., 2012) and women’s association of sex with submission (Kiefer et al., 2006) appear to be related to women’s lower sexual pleasure and greater difficulty achieving orgasms compared to men. Traditional SDS-norms have also been related to other societal problems, such as homophobia, sexism, and gender inequality (Zaikman & Marks, 2014; Zaikman et al., 2016).

Even though studies on the SDS have traditionally produced mixed results (e.g., Howell et al., 2011; Marks & Fraley, 2005; Zaikman & Marks, 2014), a recent meta-analysis yielded clear evidence for the continued existence of a traditional SDS, especially in people’s stereotyped expectations about the sexual behaviors of women and men (Endendijk et al., 2020). Yet, large variation in the degree to which people endorse SDS-norms was also found. Because of the negative consequences of traditional SDS-norms, it is important to examine a wide range of factors that could explain how these individual differences in SDS-norms develop. Most previous research examined only one or a few determinants of SDS-norms and predominantly in samples of college students, although SDS-norms were also found in adolescent and adult samples (Endendijk et al., 2020). In the current study, we examined associations between SDS-norms of Dutch adolescents (aged 16–20 years) and what parents, peers, and the media teach adolescents about appropriate sexual behavior of boys and girls (i.e., SDS-socialization). This will increase our knowledge about the relative importance of each factor, and how they covary and operate in conjunction with each other. We focus on middle and late adolescents for the following reason: they are particularly at risk for the negative consequences of the SDS, because major developments in sexuality take place in this developmental period (e.g., acquiring sexual experience, experiencing sexual debut; de Graaf et al., 2017) and they are still forming personal SDS-norms on the basis of these experiences (Zaikman & Marks, 2017). The included age range of adolescents is consistent with contemporary views on adolescence, which encompass a wider timeframe including the early twenties as well (Sawyer et al., 2018).

### A Social Norm Perspective on SDS-Socialization by Parents, Peers, and the Media

The SDS can be considered as social norm: i.e., a set of shared rules and standards that guide and/or constrain social behavior. Therefore, the SDS does not exist if it is not shared with others (Cialdini & Trost, 1998). Social norms can be transmitted by any person or context in the social sphere (Cialdini & Trost, 1998). Parents, peers, and the media are the most important socialization-agents for adolescents, but they are rarely studied together (Ballard & Morris, 1998; Epstein & Ward, 2008). According to social norm theory, adolescents are motivated to internalize the actual or perceived norms that are conveyed by these socializing-agents (Cialdini & Trost, 1998, Van de Bongardt et al., 2015), for example about the SDS. This motivation is fueled by people expecting external rewards for conforming to social norms, and sanctions for not conforming to social norms. In addition, not conforming to social norms, and the resulting deviation from a social group, is detrimental for self-esteem (Cialdini & Trost, 1998). Therefore, based on normative social influence, we expect that norms conveyed by parents, peers, and the media that reflect a traditional SDS are associated with more traditional SDS-norms in adolescents. Of the three socializing-agents, adolescents perceive the media to convey more stereotypical norms about sexual behavior of girls and boys, followed by peers and parents (Epstein & Ward, 2008). Therefore, strongest normative social influence on the SDS may be found for the media, followed by peers, and lastly by parents.

Social norm theory also distinguishes two different ways in which SDS-norms can be conveyed by parents, peers, and the media. First, SDS-norms can be transmitted in a descriptive way (i.e., descriptive norms) via the behaviors of socializing agents that are reflective of the SDS. As such descriptive norms describe appropriate sexual behavior of men and women, and provide a model for imitation. Second, SDS-norms can be transmitted in an injunctive way (i.e., injunctive norms) when socializing agents differentially approve or disapprove of the same sexual behaviors in women versus men. Injunctive norms specify how women and men should behave sexually, and motivate these behaviors by promising rewards or punishments. Both types of social norm transmission appear to play a role in adolescents’ sexual development (Van de Bongardt et al., 2015).

### SDS-Socialization by the Media

Regarding the socialization role of the media, previous studies demonstrated the separate importance of exposure to music videos of hip-hop, r&b, and rap artists (Ter Bogt et al., 2010; Van Oosten et al., 2015b), online pornography (Ortiz et al., 2016), reality television (e.g., “Geordie Shore”, Seabrook et al., 2016; Vandenbosch et al., 2015), and people’s sexy presentation on social media (Van Oosten et al., 2015a) for adolescent’s sexual behaviors, sexual attitudes, and SDS-cognitions. These media types contain considerable sexual content and behaviors that provide a descriptive norm and model for the SDS (Vandenbosch et al., 2015; Ward, 2003). For example, girls are more likely than boys to present themselves in a sexy way on social media (Vandenbosch et al., 2015) and in many TV genres and music videos men are depicted as sex-driven whereas women are depicted as sexual objects (Ward, 2003). More exposure to these descriptive norms in the media is expected to be associated with more traditional SDS-norms (Cialdini & Trost, 1998; Ward, 2003). Moreover, these media types are highly popular among adolescents. For example, over 50% of adolescents encounter pornography (Ševčíková & Daneback, 2014), with 10% being frequent users (Peter & Valkenburg, 2011). In addition, about 70% of adolescents use multiple social media platforms (Lenhart et al., 2015) and approximately 50% of teenage profiles on these platforms contain a sexualized image of the user (Crescenzi et al., 2013). The current study extends previous work by examining the relative importance of each type of media (i.e., music videos, online pornography, reality television, sexualized presentation of others on social media) for adolescents’ SDS-norms.

It is also important to examine how adolescents perceive the injunctive norms conveyed by these types of media about SDS, because these perceptions might be better predictors of SDS-norms than mere exposure (Epstein & Ward, 2008; Peter & Valkenburg, 2010). Adolescents indeed perceive the media to convey injunctive norms by differential approval of sexual behavior in men and women (e.g., “Women who have sex are sluts”, Epstein & Ward, 2008). Research is now necessary that relates these perceptions to adolescents’ personal SDS-norms.

### SDS-Socialization by Peers

Besides the media, peers can also play a role in SDS-socialization via the descriptive and injunctive norms they convey about the SDS (Van de Bongardt et al., 2015). For instance regarding descriptive norms, when adolescents perceive their male peers to be highly sexually active and their female peers to be less sexually active, this provides a model for the SDS (Cialdini & Trost, 1998). In addition regarding injunctive norms, when adolescents think their peers are more approving of boys’ having casual sex than they are of girls’ engaging in the same behavior, this conveys support for traditional SDS-norms (Cialdini & Trost, 1998). Peers indeed were found to be more accepting of male adolescents having sex or having a higher number of sexual partners than they are of female adolescents engaging in the same behaviors (Kreager & Staff, 2009; Kreager et al., 2016). There also is meta-analytic evidence that the descriptive and injunctive norms of peers about sex are associated with adolescents’ sexual activity and sexual risk behavior (Van de Bongardt et al., 2015). However, this meta-analysis did not specifically examine the influence of the norms peers convey about the SDS or the separate influence of the sexual behavior (i.e., descriptive norms) of male and female peers. In the present study, we examined how the perceived sexual behavior of male *and* female peers (i.e., descriptive norms) and the injunctive norms peers convey about the SDS are linked to adolescents’ SDS-norms.

### SDS-Socialization by Parents

With regard to parental norms, we only focus on the role of injunctive SDS-norms, because adolescents generally do not have, or do not want to have, insight in the sexual behavior of their parents (i.e., descriptive norm). Parents can convey injunctive SDS-norms in two ways First, parents can transmit information about appropriate sexual behavior of boys and girls via the rules they set for their children about dating and having sex (Mumford et al., 2016), and for using media with sexual content (Parkes et al., 2013). Such parental rules can be seen as injunctive norms as they prescribe how girls and boys *should* behave sexually, e.g., do not go on dates or sleep over with a romantic partner, or do not watch sexual media (Cialdini & Trost, 1998). When girls are sexually restricted more by these rules than boys this conveys the message that there are different norms for the sexual behavior of boys and girls. There is indeed some evidence that parents provide girls with restrictive messages regarding sexual behavior and boys with positive sex messages (Downie & Coates, 1999; Morgan et al., 2010), which has been related to more traditional SDS (Askun & Ataca, 2007).

Next to parental rules, parents can also transmit injunctive SDS-norms via their differential approval of certain sexual behaviors for boys and girls. For instance, there is ample evidence that parents are more approving of boys’ having sexual intercourse at an early age than they are of girls’ engaging in the same behavior (Connell & Elliot, 2009; Downie & Coates, 1999; Kim & Ward, 2007). Also, we know that parental beliefs about sexual permissiveness and condoms are associated with adolescents beliefs about sex and condoms (for a review, see Wright, 2009a). We only found one unpublished dissertation specifically associating injunctive SDS-norms communicated by parents to adolescents’ SDS-norms (Miller, 2012). In this study more traditional norms about the SDS communicated by mothers as well as fathers (e.g., “It is worse for a woman to sleep around than it is for a man”) were associated with more traditional SDS-norms of adolescents. More research is necessary to determine whether parents indeed transmit SDS-norms to their adolescent offspring in an injunctive way.

### A Social Learning Perspective on Gender-Specific Modeling of Socializing Agents

When examining associations between SDS-socialization conveyed by the media, peers, and parents and adolescents’ SDS-norms, it is important to take into account gender of the socializing agent and the adolescent. For example, according to social learning theory, imitation and socialization effects are supposed to be most likely for same-gender models (Bandura, 1977). Adolescents might be more likely to identify with same-gender models and same-gender models provide information about what are appropriate behaviors and beliefs for one’s own gender. Therefore, one might expect stronger associations between adolescents’ SDS-norms and peer sexual behavior, exposure to sexualized music videos, and people’s sexualized presentation on social media for same-gender models than for opposite-gender models. Some studies indeed found evidence for a same-gender modelling effect for peer influence (Lindsey, 2016) as well as media exposure (Coyne et al., 2014) on gender-stereotyped behavior. However, there are also studies finding no difference in same-gender or opposite-gender modelling of peers (Andrews et al., 2002) or the media (Coyne et al., 2016). Yet, other studies find evidence for opposite-gender modelling, with girls’ sexual attitudes being associated with viewing sexualized music videos of male artists (Van Oosten et al., 2015b). Because of these inconclusive gender-specific findings, we examined in a explorative way whether and how adolescent gender moderates the associations between SDS-norms and SDS-socialization conveyed by models in the media and male and female peers.

### Current Study

In sum, this study investigated the associations of perceived SDS-socialization conveyed by the media, peers, and parents (injunctive SDS-norms of the media, peers, and parents; exposure to sexualized music videos of male and female artists, online pornography, reality tv, and sexualized women and men on social media; sexual behavior of male and female peers; and parental rules about sex, dating, and sexual media use) with adolescents’ SDS-norms. We also explored whether perceived SDS-socialization by male and female media and peer models are associated differently with girls’ and boys’ SDS-norms. The following hypotheses were tested:

1. More exposure to media models, and female and male peers that confirm the SDS (i.e., descriptive SDS-norms) is associated with more traditional SDS-norms in adolescents;
2. More traditional injunctive SDS-norms conveyed by the media, peers, and parents are associated with more traditional SDS-norms in adolescents;
3. SDS-norms in the media are most strongly related to adolescents’ SDS-norms, followed by SDS-norms of peers, and lastly by SDS-norms of parents.

Social norms consist of different elements (Mackie et al., 2015). In the current study we focus on the social expectation element which concerns peoples’ beliefs about what others do (Mackie et al., 2015). In the context of the SDS this would entail adolescents’ expectations of the sexual behavior of women and men. It is important to mention that social norms are not necessarily congruent with one’s personal attitudes. For instance, people might believe that men take the initiative in sex more often than women because they perceive this to be the case in society. Yet, they do not necessarily have to have a negative attitude about women taking the initiative in sex. Indeed, research has shown that many people still believe the SDS to exist in society, but in their personal attitudes do not endorse the SDS (Milhausen & Herold, 2001; Rudman et al., 2013). The distinction between social norms and personal attitudes is important in social psychology, because some behaviors are more influenced by personal attitude, and other behaviors more influenced by social norms (Trafimow, 1998). Social norms about sex have been found to be more robust predictors of adolescents’ sexual behavior than personal attitudes about sex (Buhi & Goodson, 2007). Therefore, we focus on adolescents endorsement of social norms in this study. Our conceptualization of SDS-norms bears resemblance to the conceptualization by Milhausen and Herold (2001) of people’s perception of the existence of SDS in society (e.g., “Who do you think has more sexual freedom today?”) as well as Endendijk’s et al. (2020) conceptualization of SDS stereotypes (i.e., personal or socially-shared expectations about the sexual behavior of men and women).

## Methods

### Participants

Student assistants (BA and MA students in Clinical Child, Family, and Education studies) recruited classes from high schools and lower vocational schools (in Dutch: MBO) via their personal networks (e.g., own former high school, current internship organization) to participate in this study. Using information letters (provided in-person or via e-mail) the student assistants recruited 24 schools to participate in this study between November 2017 and June 2019. From each school one or two classes participated (22 schools with 1 class participating, 2 schools with 2 classes participating). The participating classes were not randomly selected, but determined by whether a teacher was willing to let the data collection take place in their class.

In total, complete data were collected from 566 adolescents aged between 16 and 20 years old (*M*_age_ = 17.17, SD = 1.00, 58% girls). In addition, 54 adolescents did not complete the entire questionnaire and were not included in this study. There were no differences between completers and non-completers on any of the background variables (ps > 0.054). Table 1 presents the background characteristics of this sample. Ethnicity of the participants was diverse and similar to the ethnic diversity in the Dutch population. In terms of educational levels, 38% of the participating adolescents were enrolled in lower secondary education, compared to 50% of adolescents in the Dutch population. Boys and girls did not differ in age (*p* = 0.14) and experience with sexual intercourse (*p* = 0.70). Boys were more often of Dutch ethnicity than girls (*χ*^2^ (6) = 27.90, *p* < 0.01, res_adj_ = 2.6). Boys were also more often enrolled in pre-university education (res_adj_ = 2.4), and less often in lower secondary education than girls (res_adj_ = − 2.3, *χ*^2^ (3) = 8.50, *p* < 0.05). Finally, boys more often had a heterosexual orientation (i.e., no romantic or sexual interest in the same gender and at least some interest in the other gender, for more information see Measures) than girls (*χ*^2^ (1) = 38.70, *p* < 0.01, res_adj_ = 6.2). All these background variables were included in further analyses.

**Table 1:** Sample characteristics

	Total sample	Girls	Boys
*n* (%)	566 (100)	331 (58)	235 (42)
Age, *M* (*SD*)	17.17 (1.00)	17.22 (1.03)	17.09 (0.95)
*Educational level, n (%)*^a^			
Lower secondary or vocational education	215 (38)	**139 (42)**	**76 (32)**
Higher secondary education	217 (38)	124 (38)	93 (40)
Pre-university education	110 (20)	**53 (16)**	**57 (24)**
Gymnasium/Grammar school	24 (4)	15 (5)	9 (4)
*Ethnicity, n (%)*			
Dutch	415 (73)	**229 (69)**	**186 (79)**
Moroccan	15 (3)	11 (3)	4 (2)
Turkish	24 (4)	**21 (6)**	**3 (1)**
Surinam	30 (5)	22 (7)	8 (3)
Asian	11 (2)	6 (2)	5 (2)
Indonesian	18 (3)	**4 (1)**	**14 (6)**
Other	53 (10)	**38 (12)**	**15 (7)**
Experience with sexual intercourse, *n* (%)	209 (37)	120 (36)	89 (38)
Heterosexual orientation, *n* (%)	425 (75)	**217 (66)**	**208 (89)**
Typical gender identity, *M*(*SD*)	2.49 (0.51)	**2.28 (0.51)**	**2.80 (0.31)**

In bold significant differences between boys and girls

^a^Educational levels are sorted from lowest to highest level

### Procedure

Participants completed an online survey (duration: approximately 45 min) via Limesurvey. The order of the questionnaires was the same for all participants (background characteristics, gender typicality, personal SDS-norms, sexual activity of peers, injunctive SDS-norms peers, exposure to sexualized music videos, reality TV, porn, and sexualized people on social media, injunctive SDS-norms media, parental restrictions, injunctive SDS-norms parents, experience with sexual intercourse, sexual orientation). This fixed order minimized the influence of parental, peer, and media SDS questionnaires on adolescents’ responses to the questionnaire assessing personal SDS-norms. Also, we chose to present sensitive topics, such as sexual orientation and sexual experience, at the end of the questionnaire. Yet, multiple items within one questionnaire were always presented randomly to participants, to reduce response sets on similarly worded questionnaires (i.e., questionnaires assessing personal SDS-norms and SDS-norms conveyed by parents, peers, and the media, see Measures). We only used previously validated questionnaires or adaptations from validated questionnaires that are commonly applied with adolescents. Participants completed the questionnaires in class under supervision of the student assistant who recruited the school to participate and received no compensation for their participation.

### Measures

#### Adolescents’ SDS-norms

We adapted the Scale for the Assessment of Sexual Standards Among Youth (SASSY, Emmerink et al., 2017) to be able to assess the complete range of possible SDS-norms from reversed to traditional. The original SASSY could not distinguish between people with reversed and egalitarian sexual standards, because both groups of people would (strongly) disagree with the items that are all worded in the direction of a traditional SDS (e.g., “I think cheating is to be expected more from boys than from girls”). Therefore, we changed the wording of the items and instead asked adolescents to indicate which gender they expected to show a certain sexual behavior more often (e.g., cheating), using a 3-point scale (0 = *both genders equally often, or neither gender*, 1 = *boys/men*, 2 = *girls/women*). A complete list of the 16 items can be found in appendix 1. We recoded the items in such a way that positive scores (+ 1) represent traditional expectations about the sexual behavior of men and women (e.g., expecting cheating more from men, and refusing sex more from women). Neutral score (0) represent egalitarian expectations about the sexual behavior of men and women (e.g., expecting cheating for both gender equally often). Negative scores (− 1) represent reversed expectations about the sexual behavior of men and women (e.g., expecting cheating more from women, and refusing sex more from men). We checked whether all items loaded onto one factor with a categorical principal component analysis (CATPCA). All items loaded onto one factor (Cronbach’s α = 0.76; see Appendix 2, Table 5for factor loadings), except for the item ‘looking attractive’. As this item did not measure sexual behaviors like the other items, we deleted this item from our measure. Recoded scores of the other 15 items were averaged to create a composite variable for personal SDS-norms.

#### SDS-Socialization

##### Injunctive SDS-norms Conveyed by the Media, Peers, and Parents

We adapted items from the SASSY (Emmerink et al., 2017) also in a different way to assess adolescents’ perceptions of the injunctive SDS-norms conveyed by the media, peers, and parents. Adolescents indicated their perceptions on statements such as: “[According to the media/My friends think/My parents think] a boy should be more knowledgeable about sex than a girl”, using a 6-point scale ranging (1 = *completely untrue* to 6 = *completely true*). Items were answered in separate questionnaires for the media, peers, and parents (see Appendix 1 for a complete list of the items). We only adapted 8 items of the SASSY with the highest factor loadings (> 0.55) to reduce the length of the questionnaire. Items were averaged in separate variables for adolescents’ perceptions of the injunctive SDS-norms conveyed by the media (Cronbach’s α = 0.92), peers (Cronbach’s α = 0.71), and parents (Cronbach’s α = 0.88).

##### Descriptive SDS-norms Media: Exposure to Sexualized Women and Men on Social Media

Adolescents reported how often, in the past 6 months, they had looked at pictures on social network sites (e.g., Facebook, Instagram) of other women/girls or men/boys in which these others presented themselves (a) with a sexy gaze, (b) with a sexy appearance, (c) scantily dressed (e.g., bathing suit or underwear), and (d) in a sexy posture (Van Oosten et al., 2015a). Response options ranged from 1 (*never*) to 7 (*multiple times a day*). Items were averaged separately for exposure to sexualized women/girls (Cronbach’s α = 0.95) and men/boys (Cronbach’s α = 0.94).

##### Descriptive SDS-norms Media: Exposure to Reality TV

On a 7-point scale (1 = *never* to 7 = *every episode*), adolescents indicated how often they watched 6 reality shows during the 6 months before the survey (e.g., MTV’s ‘‘Ex on the beach’’, MTV’s “Geordie shore”, Temptation Island) (Vandenbosch et al., 2015). We chose sexually oriented reality shows that were broadcasted before and during data collection. Items were averaged to create an exposure to reality TV variable (Cronbach’s α = 0.75).

##### Descriptive SDS-norms Media: Exposure to Online Porn

On a 7-point scale (1 = *never* to 7 = *multiple times a day*), adolescents reported on the extent to which they had intentionally watched, on the Internet, (a) pictures with clearly exposed genitals, (b) videos with clearly exposed genitals, (c) pictures in which people are having sex, (d) or videos in which people are having sex, during the last 6 months (Vandenbosch et al., 2015). Items were averaged to create an exposure to online porn variable (Cronbach’s α = 0.93).

##### Descriptive SDS-norms Media: Exposure to Sexualized Music Videos of Female and Male Artists

Adolescents indicated how often in the last 6 months they had watched music videos on the Internet or on television by 3 female (i.e., Rihanna, Nicki Minaj, Ariana Grande) and 3 male artists (i.e., Drake, Ronnie Flex, Justin Bieber) (Van Oosten et al., 2015b). We chose the artists based on three criteria: First, the artists’ music had to belong to rap, hip-hop, or R&B, which are music genres known for the highest amount of sexual content in music videos (Hansen & Hansen, 2000; Turner, 2011; Wright, 2009b), and the artists had to be known for at least some sexual content in their music videos. Second, the artists had to be popular among Dutch adolescents at the beginning of the study. Third, the artists needed to be sufficiently established in the Dutch music charts in order to remain popular over the course of the study (i.e., no “one-hit wonders”). Response options ranged from 1 (*never*) to 7 (*multiple times a day*). Items were averaged separately for exposure to sexualized music videos of female artists (Cronbach’s α = 0.95) and male artists (Cronbach’s α = 0.94).

##### Descriptive SDS-norms Peers: Sexual Activity of Male and Female Peers

We adapted a question about adolescents’ perceptions of their peers’ sexual activity (i.e., “How many of your best friends do you think have experience with intercourse?”, Fasula & Miller, 2006; Van de Bongardt et al., 2014) to 6 questions about specific sexual behaviors of male and female peers (i.e., “How many of your [female/male] friends or peers do you think [have experience with intercourse/masturbate frequently/have experience with one-night-stands]?”). Response options ranged from 1 (*none*) to 6 (*all of them*). Items were averaged separately for perceived level of sexual activity of female peers and male peers.

##### Parental Restrictions Regarding Sex and Dating or Regarding Sexual Media Use

Adolescents answered the following 3 questions about their parents restrictions regarding sex and dating: “Do your parents allow you to [sleep over with someone you are in a relationship/date with someone your parents know/date with someone your parents don’t know]?” (Mumford et al., 2016). Adolescents also indicated on two items whether their parents restricted the use of media with sexual content (“Do your parents allow you to watch series, movies or videoclips or play games [that contain a lot of nudity/with a lot of sexual activity in them]”, Parkes et al., 2013). Response options were 1 = *yes* and 0 = *no*. Items were averaged separately for parental restrictions regarding sex and dating and parental restrictions regarding sexual media use.

#### Covariates

Previous research identified the following individual characteristics of adolescents that might be associated with their SDS-norms and therefore need to be controlled for when examining the role of sexual socialization by parents, peers, and the media. First, age: older adolescents who are more likely to have experience with sexual intercourse might form more traditional SDS-norms (Zaikman & Marks, 2017). Second, with regard to adolescent gender, boys are more likely than girls to endorse traditional SDS-norms (Emmerink et al., 2016; Rudman et al., 2013). Third, people with a non-Western ethnic background endorse more traditional SDS-norms than people from a Western background (Endendijk et al., 2020; Fugère et al., 2008). Fourth, higher educational level might be associated with more egalitarian SDS-norms (e.g., Dodson & Borders, 2006; Harris & Firestone, 1998). Fifth, non-heterosexual adolescents might endorse SDS-norms less than heterosexual adolescents (Byron et al., 2017; Zaikman et al., 2016). Sixth, typicality of one’s gender identity might be associated with more traditional SDS-norms (Arthur et al., 2008; Patterson, 2012).

Therefore, adolescents reported the following background characteristics: gender (0 = *boy*, 1 = *girl*), age in years, educational level (1 = *lower secondary or vocational education*, 2 = *higher secondary education*, 3 = *pre-university education*, 4 = *gymnasium/Grammar school*), and ethnicity (1 = *Dutch*, 0 = *non-Dutch*). Adolescents also indicated whether they had experience with sexual intercourse (vaginal or anal) (1 = *yes*, 0 = *no*). Furthermore, adolescents reported on their sexual orientation (1 = *heterosexual orientation,* i.e., no romantic/sexual interest in the same-gender and at least some romantic/sexual interest in the other-gender, 0 = *non-heterosexual orientation*, all other combinations of romantic/sexual interest in the same-and other-gender). Finally, adolescents reported the typicality of their gender identity. Therefore, girls indicated whether they identified with the following labels: (1) Girly–girl, (2) Tomboy (i.e., boyish girl), (3) Androgynous (i.e., similarly boyish and girlish, or not boyish and not girlish). Boys indicated whether they identified with the following labels: (1) Boyish boy, (2) Girlish boy, (3) Metrosexual (i.e., a boy who is preoccupied with his looks), (4) Androgynous (i.e., similarly boyish and girlish, or not boyish and not girlish). Items were answered on a 3-point scale (1 = *no*, 2 = *sometimes*, 3 = *yes*). The gender identity labels were based on previous research (Ahlqvist et al., 2013; White et al., 2018). After recoding the gender-atypical items (girls: item 2 and 3, boys: item 2–4), scores were averaged into a composite variable with higher scores reflecting more typical gender identity.

### Analyses

First, we checked whether questionnaires adapted from the SASSY (SDS-norms adolescent, injunctive SDS-norms of the media, peers, and parents) measured distinct constructs, by conducting exploratory factor analyses with Maximum Likelihood and Promax rotation. See Appendix 2 for the results of these factor analyses showing a clear distinction between the different questionnaires.

Second, several descriptive analyses were used to assess associations between the study variables (i.e., Pearson correlation), gender differences on study variables (i.e., independent sample *t*-tests), differences in exposure to male and female models in the peer group or the media (i.e., paired sample *t*-tests), and differences between the media, peers, and parents in the strength of the SDS-norms they conveyed (i.e., repeated-measures ANOVA).

Third, a hierarchical multiple regression analysis was conducted to test our hypotheses with regard to the predictors of adolescents’ SDS-norms and the moderation of these associations by adolescent gender. The predictors were entered in several steps: (1) relevant covariates (inclusion of covariates was determined based on the change-in-estimate method, > 5% change criterion; Rothman et al., 2008), (2) perceived SDS-socialization by the media, peers, and parents, and (3) interactions between adolescent gender and perceived SDS-socialization by male and female media and peer models.

A priori power analyses using G*Power (Faul et al., 2009) indicated that a sample of 395 would have enough power (0.80) to detect a small effect (*f*^2^ = 0.02) of a single predictor in a multiple regression analysis with 25 predictors (α = 0.05, two-tailed). In addition, a sample of at least 520 (304 girls, 216 boys, allocation ratio N_girls_/N_boys_ = 1.41) would yield enough power (0.80) to detect a difference in slopes of 0.10 between boys and girls in a regression analysis on variables with a standard deviation of 0.5 (α = 0.05, two-tailed).

## Results

### Descriptive Statistics and Gender Differences

Table 2 displays correlations between all study variables. Most variables approached a normal distribution, except for the variables associated with exposure to sexualized music videos, reality tv, and porn. As most adolescents did not watch these types of media on a frequent basis, these variables were dichotomized (0 = *did not watch in past 6 months*, 1 = *did watch in past 6 months*). One outlier was identified on adolescents’ SDS-norms and one on the injunctive SDS-norms of peers. These outliers were winsorized (highest non-outlying number + difference between highest non-outlying number and before highest non-outlying number; Tabachnick & Fidell, 2012).

**Table 2:** Correlations between all study variables

	1	2	3	4	5	6	7	8	9	10	11	12	13
1. Adolescents’ SDS-norms													
2. Parents’ sex & dating restriction	− .07												
3. Parents’ sexual media restriction	− .04	.51**											
4. Injunctive SDS-norms parents	.18**	.13**	.17**										
5. Injunctive SDS-norms peers	.31**	.15**	.19**	.52**									
6. Sexual activity female peers (D)	− .15**	− .20**	− .21**	.00	− .02								
7. Sexual activity male peers (D)	.04	− .24**	− .23**	.07	.06	.70**							
8. Social media: sexy women (D)	.02	− .23**	− .23**	.16**	.05	.25**	.27**						
9. Social media: sexy men (D)	.04	− .07	− .08	.11**	.03	.14**	.16**	.52**					
10. Reality TV (D)	.08	− .11*	− .01	.13**	.14**	.20**	.19**	.15**	.26**				
11. Porn (D)	− .09*	− .21**	− .16**	.12**	.02	.19**	.19**	.42**	.02	.04			
12. Sexual music videos women (D)	.03	.19**	.13**	.13**	.14**	.09*	.05	.13**	.22**	.25**	− .05		
13. Sexual music videos men (D)	.01	.05	.09*	.05	.06	.14**	.11*	.18**	.20**	.27**	.00	.51**	
14. Injunctive SDS-norms media	.20**	.04	.00	.42**	.45**	.09*	.15**	.25**	.28**	.17**	.01	.14**	.10*

Abbreviation D refers to descriptive norms. * *p* < .05, ** *p* < .01

Most correlations were of small to medium size and in the expected direction. Some large positive correlations were found between the exposure to different types of sexual media, between perceived sexual activity of male and female peers, and between parental restrictions with regard to sexual media and sex/dating. Also, there were moderate to strong positive correlations between injunctive SDS-norms conveyed by the media, peers, and parents. Unexpectedly, watching more porn was associated with less traditional SDS-norms in adolescents. In addition, more parental sexual media restrictions were associated with adolescents watching *more* sexualized music videos of both male and female artists, but *less* exposure to porn and sexualized girls and women on social media.

Table 3 displays means and standard deviations for the whole sample and separately for boys and girls. Paired samples *t*-tests showed that adolescents were exposed more to sexualized girls/women than to sexualized boys/men on social media (*t*(565) = 13.86, *p* < 0.01, *d* = 0.57), but they watched more sexualized music videos of male artists than of female artists (*t*(565) =  − 3.93, *p* < 0.01, *d* = 0.16). Adolescents also perceived their male peers to be more sexually active than their female peers (*t*(565) = − 17.01, *p* < 0.01, *d* = 0.56). Adolescents perceived the media as conveying the most traditional injunctive SDS-norms, followed by their peers, and their parents (*F*(1.77, 997.65) = 153.02, *p* < 0.01, *partial η*^*2*^ = 0.21, Huynh–Feldt correction, all contrasts *p* < 0.01). There were no differences in parental restrictions with regard to sex/dating and with regard to sexual media use (*t*(565) = 0.80, *p* = 0.42).

**Table 3:** Descriptive statistics for all study variables in the whole sample and for boys’ and girls’ separately

	Total sample	Girls	Boys	*t*(df)	Cohen’s *d*
	*M* (*SD*)	*M* (*SD*)	*M* (*SD*)		
Adolescents’ SDS-norms	0.45 (0.24)	0.46 (0.25)	0.44 (0.23)	1.07 (564.00)	NS
Exposure to sexy girls/women on social media (D)	4.00 (1.72)	3.58 (1.71)	4.59 (1.57)	− 7.30 (528.41)	0.62*
Exposure to sexy boys/men on social media (D)	3.04 (1.65)	3.48 (1.61)	2.41 (1.50)	8.05 (564.00)	0.69*
Exposure to reality TV (D)	.60 (.49)	.67 (.47)	.50 (.50)	3.97 (485.01)	0.35*
Exposure to porn (D)	.55 (.50)	.33 (.47)	.86 (.35)	− 15.30 (562.23)	1.28*
Exposure to sexual music video’s women (D)	.64 (.48)	.73 (.44)	.51 (.50)	5.30 (465.30)	0.47*
Exposure to sexual music video’s men (D)	.72 (.45)	.76 (.43)	.66 (.48)	2.72 (468.91)	NS
Injunctive SDS-norms media	2.84 (1.17)	2.94 (1.20)	2.70 (1.12)	2.36 (564.00)	NS
Injunctive SDS-norms peers	2.67 (0.70)	2.69 (0.68)	2.64 (0.73)	0.95 (564.00)	NS
Sexual activity of female peers (D)	2.69 (0.89)	2.72 (0.90)	2.65 (0.89)	0.92 (564.00)	NS
Sexual activity of male peers (D)	3.24 (1.06)	3.26 (1.15)	3.22 (0.91)	0.39 (557.18)	NS
Parents’ sex & dating restrictions	.17 (.28)	.24 (.32)	.07 (.18)	8.03 (535.13)	0.63*
Parents’ sexual media restrictions	.18 (.37)	.24 (.42)	.10 (.28)	4.70 (561.43)	0.38*
Injunctive SDS-norms parents	2.12 (0.93)	2.15 (0.94)	2.09 (0.92)	0.69 (564.00)	NS

Abbreviation D refers to descriptive norms. Abbreviation NS refers to non-significant. *significant differences between boys and girls (*p* < .004)

Regarding gender differences, independent *t*-tests (*p*-level adjusted to 0.004, to account for multiple testing, see Table 3) showed that boys scored significantly higher than girls on watching sexualized girls/women on social media, and watching porn. Girls scored significantly higher than boys on watching sexualized boys/men on social media, watching reality tv, watching sexualized music videos of female artists, and parental restrictions with regard to sex/dating and sexual media. We did not find gender differences in adolescents’ SDS-norms, injunctive SDS-norms of the media, peers or parents, perceived sexual activity of female peers and male peers, and watching music videos of male artists.

### Predictors of Adolescents’ SDS-Norms

Table 4 displays results for the final hierarchical multiple regression model for adolescents’ SDS-norms. There were no indications of problematic multicollinearity between the different predictors (tolerance > 0.39, VIF < 2.56).

**Table 4:** Hierarchical multiple regression analysis predicting adolescents’ SDS-norms from individual characteristics and SDS-Socialization by the media, peers, and parents, and moderation by adolescent gender

	*B*	*SE*	*β*	*ΔR*^2^
Step 1				.09**
Age	− .02	.01	− .09*	
Gender	.05	.04	.10	
Dutch ethnicity	.10	.02	.18**	
Experience with sexual intercourse	.04	.02	.07	
Heterosexual orientation	.07	.02	.13**	
Typical gender identity	.05	.02	.09*	
Step 2				.16**
Exposure to sexy girls/women on social media (D)	.02	.01	.13	
Exposure to sexy boys/men on social media (D)	< .01	.01	.11	
Exposure to reality TV (D)	< .01	.02	< .01	
Exposure to porn (D)	− .03	.02	− .07	
Exposure to sexual music video’s women (D)	.07	.03	.14*	
Exposure to sexual music video’s men (D)	− .07	.04	− .12	
Injunctive SDS-norms media	.02	.01	.09*	
Injunctive SDS-norms peers	.09	.02	.26**	
Sexual activity of female peers (D)	− .06	.02	− .21**	
Sexual activity of male peers (D)	.04	.02	.17	
Parents’ sex & dating restrictions	− .01	.02	− .02	
Parents’ sexual media restrictions	− .02	.02	− .06	
Injunctive SDS-norms parents	.01	.01	.03	
Step 3				.02*
Gender × Exposure to sexy girls/women on social media	− .05	.02	− .26*	
Gender × Exposure to sexy boys/men on social media	.03	.02	.15	
Gender × Exposure to sexual music videos women	− .10	.05	− .20*	
Gender × Exposure to sexual music videos men	.07	.05	.15	
Gender × Sexual activity of female peers	− .03	.03	− .09	
Gender × Sexual activity of male peers	.01	.03	.02	
Total *R*^2^ after step 3				.28**

Table displays regression coefficients of the final model. Abbreviation D refers to descriptive norms. *p < .05, **p < .01

#### Covariates

Including relevant covariates of adolescents in Step 1 lead to a significant model (*F*(6, 559) = 9.69, *p* < 0.01). The model explained 9% of the variance in adolescents’ SDS-norms. Having experience with sexual intercourse and gender were not significantly associated with adolescents’ SDS-norms. However, younger age, Dutch ethnicity, having a heterosexual orientation, and a gender typical identity were associated with more traditional SDS-norms.

#### Perceived SDS-socialization by the Media, Peers, and Parents

In Step 2 all the sexual socialization variables were added. This lead to a significant model (*F*(19, 546) = 9.81, *p* < 0.01) and a significant 16% increase in the explained variance in adolescents’ SDS-norms. More traditional injunctive SDS-norms conveyed by the media and peers (as perceived by adolescents) were associated with more traditional SDS-norms of adolescents. In addition, more exposure to sexualized music videos of female artists was related to more traditional SDS-norms in adolescents (this main effect was subsumed by an interaction with adolescent gender, see Step 3). Finally, less sexually active girls in an adolescent’s peer group were associated with more traditional SDS-norms in adolescents. Adolescents’ SDS-norms were not predicted by exposure to sexualized people on social media, watching reality TV, porn, or sexualized music videos of male artists, sexual activity of boys in an adolescent’s peer group, parents’ sex, dating, and sexual media restrictions, and parents’ injunctive SDS-norms.

#### Interactions Between Adolescent Gender and SDS-socialization by Male and Female Media and Peer Models

**Fig. 1:**
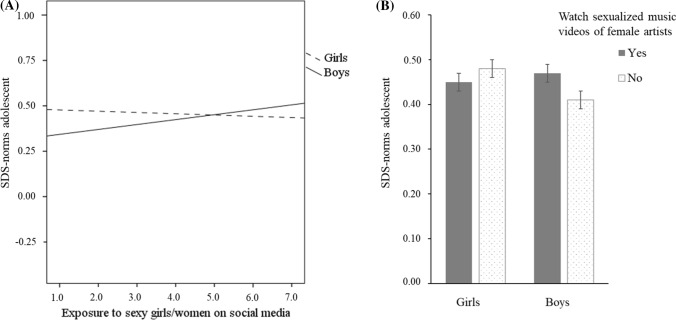
Adolescent gender interacting with exposure to sexy girls/women on social media (**a**) and watching sexual music videos of female artists (**b**) in predicting adolescents’ SDS-norms. *Note*. Error bars represent standard errors of the means

In Step 3 we added the 6 interactions of adolescent gender with exposure to sexualized music videos by male and female artists, exposure to sexualized women and men on social media, and perceived sexual behavior of male and female peers. This lead to a significant model (*F*(25, 540) = 8.19, *p* < 0.01) and a significant 2% increase in the explained variance in adolescents’ SDS-norms. This final model explained 28% of the variance in adolescents’ SDS-norms. Only the interactions of gender with exposure to sexualized girls/women on social media and watching sexualized music videos of female artists were significant. The interaction effects are shown in Fig. 1. For boys exposure to sexualized girls/women on social media was associated with more traditional SDS-norms (*β* = 0.16, *p* < 0.05), whereas for girls this was associated with less traditional SDS-norms (*β* = -0.22, *p* < 0.05; see Fig. 1a). Similarly, boys who watched sexualized music videos of female artists endorsed more traditional SDS-norms than boys who did not watch these music videos (*t*(233) = − 1.99, *p* < 0.05, *d* = 0.26; see Fig. 1b). Girls who watched or did not watch sexualized music videos of female artists did not significantly differ in their SDS-norms (*t*(329) = 1.03, *p* = 0.31).

Even though we were primarily interested in testing the same-gender modeling hypothesis, we also checked whether associations between the other SDS-socialization variables (i.e., parents’ restrictions regarding sex and dating or sexual media, injunctive SDS-norms of the media, peers, and parents, exposure to reality TV or porn) and SDS-norms were different for girls and boys. Therefore, we used Fisher’s *z*-test to compare beta’s from separate regression analyses (including all SDS-socialization variables) of girls and boys. There were no differences between girls and boys for associations with any of the other SDS-socialization variables (*z*s < 1.47, *p*s > 0.05).

## Discussion

In this study we examined associations between adolescents’ SDS-norms with several individual characteristics and SDS-socialization conveyed by the media, peers, and parents. We also explored whether associations between perceived SDS-socialization by male and female models in the media and peer context and adolescents’ SDS-norms differed between boys and girls. We found partial support for our first hypothesis because more exposure to female peers, but not to media models or male peers, who confirm the SDS (i.e., descriptive SDS-norms) was associated with more traditional SDS-norms in adolescents. The second hypothesis was also partly confirmed because more traditional injunctive SDS-norms conveyed by the media and peers, but not by parents, were associated with more traditional SDS-norms in adolescents. The third hypothesis was fully supported by the findings that SDS-norms in the media are most strongly related to adolescents’ SDS-norms, followed by SDS-norms of peers, and not by SDS-norms of parents.

### Perceived SDS-socialization by the Media, Peers, and Parents

Regarding SDS-socialization, more traditional injunctive SDS-norms conveyed by the media and peers, as well as less perceived sexual activity of female peers (i.e., descriptive norms), were associated with more traditional SDS-norms in adolescents. Apparently, aspects of SDS-socialization by the media and peers, and not by parents, were associated with adolescents’ SDS-norms. This is consistent with the diminishing influence of parents on adolescent development, and the increased influence of peers and the media, especially in the domain of sexual development (L’Engle & Jackson, 2008; L’Engle et al., 2006; Ragsdale et al., 2014; Scull et al., 2018). Also, adolescents perceive the media to convey more stereotypical norms about sexual behavior of girls and boys, followed by peers and parents (Epstein & Ward, 2008). Furthermore, adolescents’ might expect more rewards and less sanctions for not conforming to SDS-norms from peers than from parents (Kreager & Staff, 2009). Because of these processes, adolescents might be more motivated to internalize the perceived descriptive and injunctive norms that are conveyed by peers and the media about the SDS in their personal SDS-norms (Cialdini & Trost, 1998). In addition, the finding that both descriptive and injunctive SDS-norms of socializing agents are associated with adolescents’ SDS-norms indicate that both modelling/imitation and reward/punishment processes are underlying the transmission of SDS-norms (Cialdini & Trost, 1998).

Regarding SDS-socialization by the media, it appeared that the perceived traditional norms and messages conveyed by the media about the SDS (i.e., injunctive norms) are associated with adolescents’ SDS-norms, whereas mere exposure to descriptive SDS models in the media is not (at least not in the group of adolescents as a whole). This finding fits with research showing that the perceptions about media content might be better predictors of personal attitudes and behavior than mere exposure (Epstein & Ward, 2008; Peter & Valkenburg, 2010).

Regarding SDS-socialization by peers, as expected, adolescents perceived their male peers to be more sexually active than their female peers, which provides a descriptive model for the SDS. Consequently, less perceived sexual activity of female peers was associated with more traditional SDS-norms in both boys and girls. Interestingly, only the descriptive norm conveyed by the sexual behavior of female peers, and not of male peers, was associated with adolescents’ SDS-norms. It might be the case that when the sexual behavior of female peers does not conform to the SDS this might be more salient for adolescents than when the sexual behavior of male peers does not conform to the SDS. Therefore, the sexual behavior of female peers might be more informative for adolescents’ SDS-norms. Some support for this reasoning is provided by research showing that women who violate the SDS are evaluated less positively than men who violate the SDS (Young et al., 2016).

Not only the perceived sexual behavior of male and female peers, but also the injunctive SDS-norms conveyed by peers were related to adolescents’ SDS-norms. When peers are more approving of boys’ having casual sex they are of girls’ engaging in the same behavior, this conveys support for traditional SDS-norms. Our findings fit with meta-analytic evidence that both injunctive and descriptive norms of peers about sex are associated with adolescents’ sexual outcomes (Van de Bongardt et al., 2015). However, we extend this research by showing that the perceived sexual behavior of male *and* female peers, as well as the specific *SDS*-attitudes of peers are associated with adolescents’ SDS-norms. In future research it would be interesting to examine whether *perceived* and *actual* behaviors and attitudes of social referents have different effects on adolescents SDS-cognitions and behavior (Cialdini & Trost, 1998).

The lack of association between perceived parental rules about sex, dating, and sexual media use and adolescents’ SDS-norms might be due to a lack of insight of adolescents into the actual rules parents set in this regard (Rogers et al., 2006). Future research could examine whether parent-reported rules about sex, dating, and sexual media use, but also of parents’ self-reported injunctive SDS-norms are better predictors of adolescents’ SDS-norms. However, it is also possible that whether adolescents actually follow or ignore parental rules about dating and sex (Hovell et al., 1994) or parental enforcement of these rules (Dittus et al., 2015) are more important predictors of adolescents sexual behavior and cognitions than adolescents perceptions of parental rules about dating and sex. Furthermore, the different ways in which parents communicate about sex with girls and boys might also be important predictors of adolescents’ SDS-norms. For instance, girls received more restrictive sex messages from their parents, whereas boys received more positive-sex messages (Flores & Barroso, 2017; Morgan et al., 2010) which confirms traditional SDS-norms. A final reason for the lack of effects found for parents’ injunctive SDS-norms is that parents might not convey to their adolescents the rather explicit messages we examined (e.g., “Boys are more entitled to sexual pleasure than girls”). Parents instead might be more subtle in their messages (e.g., “It is more appropriate for boys than for girls to have sexual intercourse at an early age”).

### Gender-Specific Modeling of Socializing Agents in the Media and the Peer Group

First, no gender-specific associations were found between adolescents’ SDS-norms and the perceived sexual behavior of male and female peers. Thus, for boys and girls the perceived sexual behavior of both male and female peers are important in relation to their personal SDS-norms. Second, only for boys, exposure to sexualized girls/women on social media and sexualized music videos of female artists, as well as injunctive SDS-norms conveyed by the media were associated with more traditional SDS-norms. Both findings for peer and media models do not completely fit with social learning theory’s propositions about the importance of same-gender modelling (Bandura, 1977). For boys’ SDS-norms, opposite-gender models in the media are apparently more informing. This could be because sexualized music videos of female artists have been found to contain more sexual-objectification (i.e., portray women as sex objects) than music videos of male artists (Aubrey & Frisby, 2011). Women are also more likely to engage in sexual self-objectification on social media than men (Hall et al., 2012; Manago et al., 2008). Consequently, exposure to high levels of sexual objectification in sexualized music videos of female artists or in female social media profiles are related to rape myth stereotypes (Kistler & Lee, 2010) and stereotyped beliefs about women’s sexual behavior (Aubrey et al., 2011). These associations are found primarily for men, possibly because women might be more offended by the sexualized presentation of women in the media (Ward, 2016). For boys, exposure to sexual objectification of female bodies in the media may prime a schema of women as sexual objects and associated expectations of female sexual reactiveness and submissiveness (Aubrey et al., 2011). On the other hand, for girls exposure to sexualized females on social media might signal the sexual autonomy of women which does not fit with traditional SDS-norms. It should be mentioned that the modeling effect in music videos is difficult to interpret as music videos of female artists often also include men, and videos of male artists often include women. Therefore, it is unclear which models actually inform boys’ SDS-norms.

Interestingly, associations between sexual media exposure and boys’ SDS-norms were particularly found for social media and sexualized music videos, and not for pornography or reality tv. Thus, social media and sexualized music videos might be relatively more important than pornography and reality tv in relation to adolescents’ SDS-norms. The importance of sexual female models on social media is not surprising considering that on social media adolescents are also exposed to feedback of other people on the sexual appearance of girls/women, in the form of for example slut-shaming. As such social media might combine SDS-socialization by two socializing agents, i.e., peers and the media. In addition, social media feature “real” peers that are easy to identify with, instead of actors such as in porn (Ward, 2016). Yet, the lack of association with exposure to “real” models in reality tv shows might be due to the low frequency of adolescents’ watching the shows that were examined in this study. More extensive analysis of the sexual content of a wide range of reality tv programs is necessary, as well as studies examining how exposure to sexual content featuring reality characters relates to viewers sexual behavior and cognitions. The importance of sexualized music videos can be explained by their construction around common, simple social events and themes that can be easily represented in memory in the form of scripts about the sexual behavior of women and men (Hansen, 1989).

### Individual Characteristics of Adolescents

For the covariates, younger age, Dutch ethnicity, having a heterosexual orientation, and a gender typical identity were associated with more traditional SDS-norms. We will only discuss unexpected effects or effects that might have clear practical implications. First, the finding that younger adolescents in our sample endorsed more traditional SDS-norms suggests that pressure to conform to gender norms might be higher earlier in adolescence than later in adolescence (Basow & Rubin, 1999; Hill & Lynch, 1983). Therefore, interventions aimed at targeting SDS-norms should commence early in adolescence. Second, the finding for Dutch ethnicity might be obscured because the group of adolescents with a non-Dutch background was highly diverse in terms of ethnicity.

### Practical Implications

Our findings have some practical implications for future interventions or sex education programs. First, our findings signal the need for incorporation of topics related to the SDS in sex education, as adolescents hold stereotypical expectations about the sexual behavior of men and women that might hamper their sexual development. Currently, many Western sex education programs in schools do not include SDS-related topics such as slut-shaming, sexual coerciveness of men, or sexual pressure exerted on men (e.g., de Graaf et al., 2017; Hall et al., 2019). Second, the relative importance of media and peers in the transmission of SDS-norms to adolescents suggests that interventions should focus on increasing adolescents’ resilience to the normative influence of the media and peers. Considering that we focused primarily on adolescents’ *perception* of the SDS-norms conveyed by the media and peers, resilience might be fostered by providing adolescents with nuanced information about girls’ and boys’ healthy and realistic engagement in sexual behavior. In addition, adolescent’s resilience to sexual media, in particular sexualized social media and music videos, might be increased by informing them about the unrealistic sexual standards set in the media. Providing adolescents with accurate information about the prevalence of SDS-related behaviors (i.e., descriptive norms) as well as attitudes (i.e., injunctive norms) among peers and in the media, might lead to a reduction in misperceptions of the sexual behavior of women and men (Brechwald & Prinstein, 2011; Prentice & Miller, 1996). This approach has proven to be effective in the prevention of adolescent risk behavior (Prentice, 2008). Finally, as we found that most predictors of adolescents’ SDS-norms were the same for boys and girls, the content of interventions and sex education targeting the SDS could be the same for both genders. Providing the same content to boys and girls might be particularly important as it has been argued that different programs for boys and girls might maintain sexual stereotypes (Szirom, 2017).

### Limitations and Future Directions

Our findings must be viewed in light of some limitations. Because of the correlational design of this study, we were not able to determine the direction of effects in the association between adolescents’ SDS-norms and SDS-socialization by the media, peers, and parents. Therefore, we were not able to conclude whether our results reflect selection effects (e.g., adolescents with traditional SDS-norms hanging out with peers with similar normative beliefs, or choosing to watch media with sexual content), socialization effects (e.g., adolescents internalize similar SDS-norms as their peers, or engage in similar sexual behaviors as models in the media), or both. Long-term longitudinal studies examining bidirectional associations between SDS-socialization and adolescents’ SDS-norms can provide further clarity on the relative importance of selection and socialization effects.

Further, we only used self-report measures to assess adolescents’ SDS-norms and their correlates, which increases the risk of social desirability in responding and of shared-method variance. However, self-reports are appropriate to employ, considering our focus on *perceived* SDS-socialization practices. Moreover, correlations between self-reported aspects of personal SDS-norms and socialization could be seen as an indication of the importance of cognitive schemas in people’s representations of the SDS. In addition, adolescents could complete the online survey anonymously and there was considerable variation in adolescents’ SDS-norms which was not related to educational level, which suggests that social desirability issues were unlikely to be present.

In addition, the factor loadings of the questionnaire assessing adolescent SDS-norms were on the low side. This is most likely due to the 3-point response we used. Scales with few response options are known to be related to lower reliability and reduced association between items (e.g., Lozano et al., 2008). Future research with our SDS-norm questionnaire should preferably use a 5-point response scale (i.e., 1 = men/boys much more, 2 = boys/men somewhat more, 3 = both genders equally often, 4 = girls/women somewhat more, 5 = girls/women much more) and examine whether our findings can be replicated.

Finally, in our assessment of injunctive SDS-norms of peers we did not differentiate between male and female peers, even though female and male peer norms might be differentially related to adolescents’ SDS norms. Heterosexual adolescents might be more motivated to internalize the injunctive SDS-norms of opposite-gender peers compared to the norms of same-gender peers to come across as a desirable sexual partner. Future research could address this possibility.

### Conclusion

In sum, our findings imply that perceived SDS-socialization by the media and peers play a role in adolescents’ expectations about the sexual behavior of women and men. Adolescent girls and boys were equally susceptible to the SDS-socialization by male and female peers, but boys were more susceptible to exposure to media with sexual content, in particular sexualized music videos of female artists and sexualized presentation of women on social media. An important next step to take is to longitudinally examine associations between SDS-socialization, adolescents’ SDS-norms, and adolescents’ own sexual behavior. This will provide essential knowledge about the developmental processes underling SDS endorsement and enactment. Regarding practical implications, our findings show that intervention efforts targeting SDS-norms should commence early in adolescence and focus on increasing adolescents’ resilience to the normative influence of the media and peers. Such intervention efforts might contribute to adolescents’ endorsement of egalitarian sexual standards for women and men, and freedom for both women and men to express their sexuality in the way they desire.

## Appendix 1: Overview of Items in the Questionnaires Assessing SDS-norms

### Adolescent Questionnaire

Who do you expect to more often show the following behaviors?

1. Refusing sex.
2. Taking the initiative in sex.
3. Having different sexual partners at the same time.
4. Acting in a more reserved way concerning sex.
5. Having sex without love.
6. Having a lot of knowledge about sex.
7. Finding sex important.
8. Taking the dominant role in sex.
9. Using sexually explicit talk.
10. Applying some pressure on another person to get what one wants sexually.
11. Keeping one’s virginity until marriage.
12. Having unusual sexual desires.
13. Looking attractive.
14. Cheating.
15. Act as if one is sexually active, even if it is not true.
16. Frequent masturbating.

### Questionnaires Assessing Perceived Injunctive SDS-norms Conveyed by Parents, Peers, and the Media

1. [My parents think/My friends think/According to the media] boys are more entitled to sexual pleasure than girls.
2. [My parents think/My friends think/According to the media] it is more appropriate for a boy than for a girl to masturbate frequently.
3. [My parents think/My friends think/According to the media] it is important for a boy to act as if he is sexually active, even if it is not true.
4. [My parents think/My friends think/According to the media] a boy should be more knowledgeable about sex than a girl.
5. [My parents think/My friends think/According to the media] girls should act in a more reserved way concerning sex than boys.
6. [My parents think/My friends think/According to the media] cheating is to be expected more from boys than from girls.
7. [My parents think/My friends think/According to the media] it is normal for boys to take the dominant role in sex.
8. [My parents think/My friends think/According to the media] Sex is more important for boys than for girls.

## Appendix 2: Factor Analyses Showing Distinction Between Different Questionnaires

See Tables 5, 6, 7, 8, 9 and 10.

**Table 5:** Factor loadings for CATPCA on items of the questionnaire assessing adolescent SDS-norms

	Factor
SDSstereo1	.546
SDSstereo2	.534
SDSstereo3	.402
SDSstereo4	.186
SDSstereo5	.562
SDSstereo6	.563
SDSstereo7	.416
SDSstereo8	.376
SDSstereo9	.385
SDSstereo10	.465
SDSstereo11	.470
SDSstereo12	.588
SDSstereo13	.517
SDSstereo14	.527
SDSstereo15	.378
SDSstereo16	.388

**Table 6:** Factor loadings of CATPCA with promax rotation for distinction between adolescents’ SDS-norms and injunctive sds-norms conveyed by parents

	Factor 1	Factor 2
SDSstereo1	− .009	**.555**
SDSstereo2	.030	**.532**
SDSstereo3	.013	**.396**
SDSstereo5	− .107	**.592**
SDSstereo6	.016	**.563**
SDSstereo7	− .013	**.420**
SDSstereo8	.046	**.358**
SDSstereo9	.070	**.359**
SDSstereo10	.066	**.436**
SDSstereo11	− .091	**.498**
SDSstereo12	.042	**.587**
SDSstereo13	.042	**.498**
SDSstereo14	− .022	**.535**
SDSstereo15	− .017	**.385**
SDSstereo16	− .009	**.384**
SDSparent1	**.712**	.034
SDSparent2	**.850**	− .038
SDSparent3	**.720**	.125
SDSparent4	**.817**	− .084
SDSparent5	**.824**	− .020
SDSparent6	**.774**	.101
SDSparent7	**.832**	− .009
SDSparent8	**.828**	− .067

Factor loadings in bold reflect loadings higher than .30

**Table 7:** Factor loadings of CATPCA with promax rotation for distinction between adolescents’ SDS-norms and injunctive sds-norms conveyed by peers

	Factor 1	Factor 2
SDSstereo1	− .116	**.605**
SDSstereo2	.025	**.531**
SDSstereo3	.055	**.374**
SDSstereo5	− .081	**.593**
SDSstereo6	.027	**.556**
SDSstereo7	.001	**.406**
SDSstereo8	.116	**.299**
SDSstereo9	.085	**.328**
SDSstereo10	.060	**.434**
SDSstereo11	− .029	**.470**
SDSstereo12	− .079	**.642**
SDSstereo13	.101	**.464**
SDSstereo14	.009	**.522**
SDSstereo15	− .047	**.398**
SDSstereo16	− .015	**.386**
SDSpeer1	**.681**	− .021
SDSpeer2	**.769**	− .189
SDSpeer3	**.558**	.216
SDSpeer4	**.808**	− .090
SDSpeer5	**.545**	.239
SDSpeer6	**.731**	.083
SDSpeer7	**.749**	− .134
SDSpeer8	**.642**	.119

Factor loadings in bold reflect loadings higher than .30

**Table 8:** Factor loadings of CATPCA with promax rotation for distinction between adolescents’ SDS-norms and injunctive sds-norms conveyed by the media

	Factor 1	Factor 2
SDSstereo1	.070	**.516**
SDSstereo2	.107	**.496**
SDSstereo3	− .009	**.406**
SDSstereo5	− .009	**.561**
SDSstereo6	− .042	**.583**
SDSstereo7	.052	**.398**
SDSstereo8	− .122	**.426**
SDSstereo9	− .080	**.420**
SDSstereo10	.040	**.452**
SDSstereo11	− .029	**.478**
SDSstereo12	.099	**.557**
SDSstereo13	− .060	**.540**
SDSstereo14	− .050	**.547**
SDSstereo15	.108	**.340**
SDSstereo16	− .030	**.400**
SDSmedia1	**.748**	− .051
SDSmedia2	**.775**	− .006
SDSmedia3	**.842**	− .034
SDSmedia4	**.810**	− .006
SDSmedia5	**.839**	.023
SDSmedia6	**.771**	.028
SDSmedia7	**.799**	.004
SDSmedia8	**.833**	− .002

Factor loadings in bold reflect loadings higher than .30

**Table 9:** Factor loadings of CATPCA with promax rotation for distinction between adolescents’ SDS-norms and perceived sexual activity of peers

	Factor 1	Factor 2
SDSstereo1	**.553**	.067
SDSstereo2	**.542**	.161
SDSstereo3	**.401**	.026
SDSstereo5	**.558**	.014
SDSstereo6	**.565**	− .057
SDSstereo7	**.421**	.054
SDSstereo8	**.373**	− .084
SDSstereo9	**.379**	− .081
SDSstereo10	**.459**	− .051
SDSstereo11	**.468**	.032
SDSstereo12	**.595**	.126
SDSstereo13	**.509**	− .140
SDSstereo14	**.527**	− .022
SDSstereo15	**.378**	.005
SDSstereo16	**.385**	− .240
PeerSexActive1	.014	**.755**
PeerSexActive2	− .118	**.683**
PeerSexActive3	− .120	**.680**
PeerSexActive4	.097	**.834**
PeerSexActive5	.092	**.662**
PeerSexActive6	.010	**.739**

Factor loadings in bold reflect loadings higher than .30

**Table 10:** Factor loadings for CATPCA with promax rotation for distinction between injunctive SDS-norms conveyed by parents. peers. and the media

	Factor 1	Factor 2	Factor 3
SDSparent1	.106	**.732**	− .103
SDSparent2	− .059	**.828**	.075
SDSparent3	.127	**.731**	− .078
SDSparent4	− .174	**.803**	.132
SDSparent5	− .064	**.852**	.003
SDSparent6	.117	**.803**	− .109
SDSparent7	.095	**.815**	− .058
SDSparent8	− .106	**.782**	.142
SDSpeer1	.017	.030	**.645**
SDSpeer2	− .163	− .065	**.841**
SDSpeer3	.183	− .033	**.546**
SDSpeer4	− .129	.026	**.838**
SDSpeer5	.255	− .058	**.519**
SDSpeer6	.158	.011	**.667**
SDSpeer7	− .067	.011	**.753**
SDSpeer8	.191	.079	**.525**
SDSmedia1	**.633**	.025	.138
SDSmedia2	**.857**	− .038	− .120
SDSmedia3	**.777**	.049	.029
SDSmedia4	**.705**	.078	.105
SDSmedia5	**.814**	.015	.039
SDSmedia6	**.860**	− .075	− .059
SDSmedia7	**.794**	.043	− .022
SDSmedia8	**.851**	− .028	− .007
